# Case Report: endoscopic microvascular decompression for trigeminal neuralgia in a centenarian: a landmark case with 5-year follow-up

**DOI:** 10.3389/fsurg.2025.1537318

**Published:** 2025-08-11

**Authors:** Xuhao Fang, Feng Tang, Yao Deng, Yue Zhou, Weidong Gu, Renling Mao

**Affiliations:** ^1^ Department of Neurosurgery, Huadong Hospital, Fudan University, Shanghai, China; ^2^ Department of Anesthesiology, Huadong Hospital, Fudan University, Shanghai, China

**Keywords:** trigeminal neuralgia, centenarian, microvascular decompression, neuroendoscope, case report

## Abstract

**Background:**

Endoscopic Microvascular Decompression (E-MVD) is a well-established effective treatment option for trigeminal neuralgia (TN). However, its application in ultra-elderly patients, particularly centenarians, has never been documented.

**Case Description:**

A centenarian with TN for five years presented to our hospital in January 2020. Her symptoms had progressively worsened over the preceding three months. Despite extreme age, her organ function remained relatively preserved based on comprehensive physical and laboratory examinations. E-MVD was performed, and the patient experienced immediate pain relief without any neurological dysfunction. During five years of follow-up, there was no recurrence of symptoms; the patient remains symptom-free at 105 years of age.

**Conclusion:**

This case study presents the successful use of E-MVD in a centenarian patient suffering from TN, highlighting its potential as a viable treatment option even in this advanced age group. Further studies are needed to confirm these findings and explore the generalizability of this approach.

## Introduction

Trigeminal Neuralgia (TN) is a chronic pain disorder that affects the head and face, with an incidence of 4.3 out of every 100,000 individuals in the general population, rising to 25.6 per 100,000 annually among those over 70 years old (1, 2). Microvascular Decompression (MVD) is a well-established and effective treatment for TN (3, 4). Recently, endoscopic Microvascular Decompression (E-MVD) has gained popularity due to its mini-invasiveness and established efficacy (5–8). Despite its benefits, the application of E-MVD in ultra-elderly patients, particularly centenarians, has not been previously reported. A targeted literature search of PubMed and Embase conducted on November 30, 2024, using relevant terms confirmed no prior published cases involving patients aged 100 years or older undergoing this procedure. This report presents the first successful application of E-MVD in a centenarian with TN. The reporting of this case was prepared in adherence to the CARE checklist (https://www.care-statement.org/checklist).

## Case report

A centenarian with TN for five years presented to our hospital in January 2020. After a detailed history-taking, it was revealed that the pain began at age 95, manifesting as typical intermittent, striking pain with trigger points. Due to advanced age, no neurosurgical plans were considered for her then. Therefore, she began medical treatment with carbamazepine, and initially, the medication was effective. However, gradually, it became ineffective despite attempts to increase the dose of carbamazepine and the addition of other drugs such as pregabalin (PGB), gabapentin (GP), and lamotrigine (LTG). The pain persisted, and even powerful opioid painkillers failed to provide relief. Over the past three months, her symptoms had worsened, persisting for almost 24 h every day. During these three months, the patient experienced significant distress, becoming unable to eat or sleep, and even considering suicide. Following the visit, she was admitted for further evaluation and treatment.

**Figure 1:**
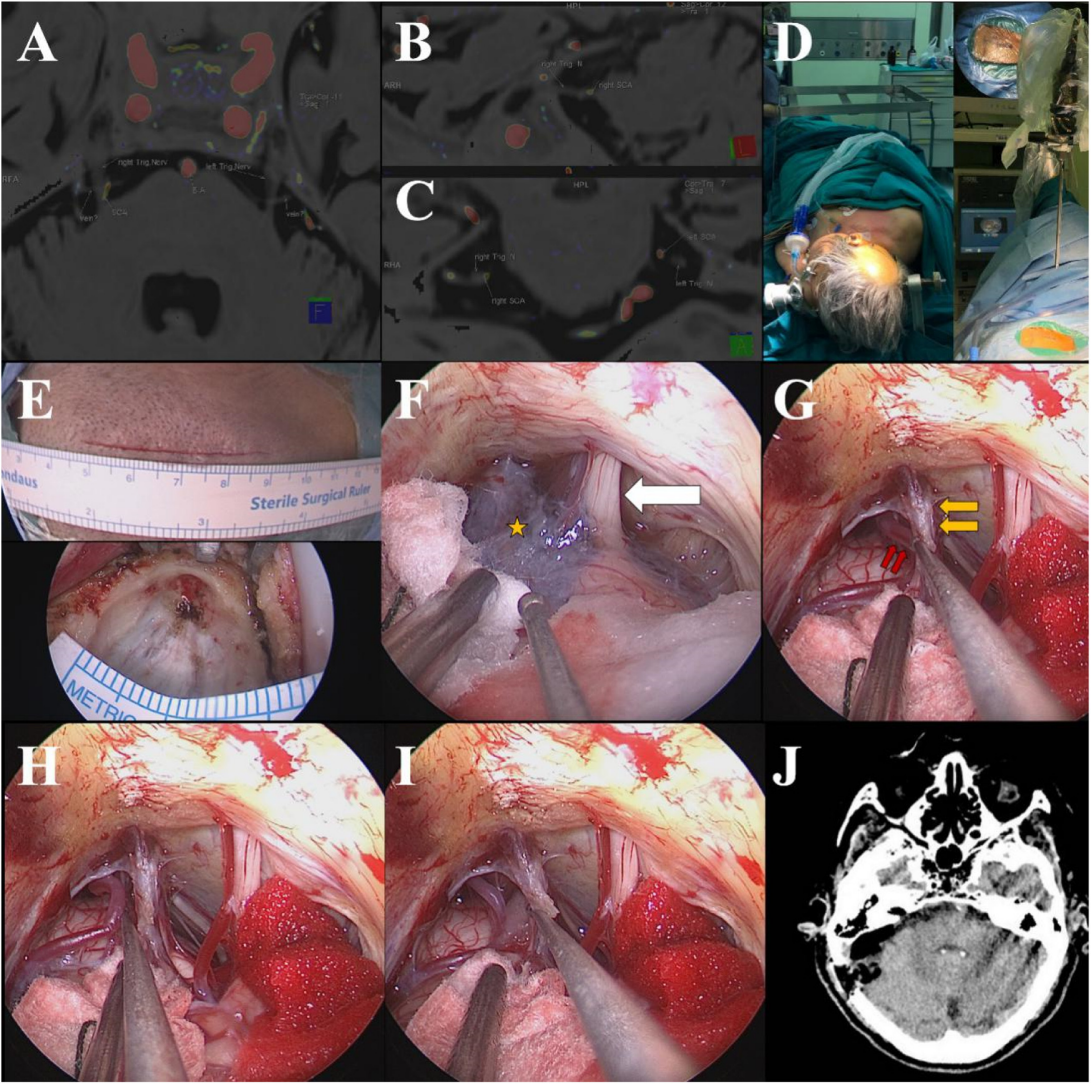
MRTA clearly showed the relationship between the right REZ of the trigeminal nerve and the right SCA **(A–C)**; supine position after the general anesthesia and the neuroendoscope fixed with a pneumatic arm **(D)**; the incision [**(E)**, upper] and craniectomy [**(E)**, lower]; endoscopic visions showed the thickening of the arachnoid membrane (yellow star) in the cistern of CPA and the cranial nerve VII/VIII (white arrow) **(F)**; the right SCA (the double red arrows) and the right trigeminal nerve in front of the right SPV (double yellow arrows) **(G)**; the right SCA being dissected away from the right REZ of the trigeminal nerve **(H)**; Teflon spacer being interposed between the REZ and the SCA **(I)**; head CT scan immediately after the operation **(J)**.

The patient was in such pain that she refused any touching of the right side of her face, with a Numerical Rating Scale (NRS) score of 10 and Barrow Neurological Institute Pain Intensity (BNI) Score of 5, indicating the worst possible pain. Magnetic Resonance Tomographic Angiography (MRTA) revealed that the right trigeminal nerve root was compressed by the right superior cerebellar artery (SCA) (Figures 1A–C). Upon physical examination, the patient had a normal body temperature of 36.7°C and a blood pressure of 140/80 mmHg, with the systolic pressure being within the high-normal range. Furthermore, chest CT scans and lung function tests indicated relatively good respiratory function. Electrocardiogram and cardiac ultrasound examinations confirmed relatively good cardiac function. Blood tests revealed healthy liver and kidney function, and the combination of blood tests and physical examination suggested moderate nutritional status. Cognitive evaluation demonstrated a Mini-Mental State Examination (MMSE) score of 21/30, indicating preserved baseline cognitive function. After thorough communication with the patient and their family members, the option of an E-MVD surgery was presented. The surgery was scheduled for the patient in the morning of the fifth day after her admission.

The patient's surgery was performed under general anesthesia with intravenous administration and tracheal intubation. Drugs including sufentanil, etomidate, and rocuronium were used for anesthesia induction, followed by remifentanil and desflurane for maintenance. The dosage of the drugs was adjusted based on the patient's vital signs and the depth of anesthesia to ensure stability and prevent overdose.

The patient was positioned supine with the right shoulder elevated and the head slightly tilted to the left (Figure 1D). A posterior sigmoid keyhole approach was utilized, and a 2.5 cm diameter craniectomy was performed through a 5 cm straight retroarticular incision (Figure 1E). The dura mater and arachnoid membrane were opened, cerebrospinal fluid was released slowly, and the cerebellopontine angle (CPA) space was exposed for the insertion of the neuroendoscope (zero degree, 4 mm, Karl Storz), which was stabilized using a pneumatic arm. Under endoscopic visualization, thickening of the CPA arachnoid membrane was observed (Figure 1F). The arachnoid membrane was meticulously dissected using microscopic scissors to expose the superior petrosal vein (SPV) and the trigeminal nerve region. This dissection also relieved tension on the superficial aspects of the facial and vestibular nerves. The right trigeminal nerve was located anterior to the right SPV and partially obscured by the SPV under endoscopic view (Figure 1G), nevertheless, the compression of the root exit/entry zone (REZ) of the right trigeminal nerve by the right SCA was identified and the SCA was displaced using a delicately curved neurodissector (Figure 1H). A Teflon spacer was carefully placed to elevate the SCA towards the tentorium until a satisfactory position was achieved, effectively relieving compression on the REZ (Figure 1I). After confirming no bleeding at the operative site, the neuroendoscope was withdrawn. The dura mater was closed in a watertight fashion, the bone flap was replaced, and the scalp was sutured using an intradermal closure technique.

During the procedure, the patient experienced a transient increase in blood pressure and a decrease in heart rate upon the surgical team's approach to the trigeminal nerve root. However, these vital sign fluctuations resolved promptly upon cessation of manipulation near the nerve. Postoperative anesthesia recovery was uneventful, the endotracheal tube was removed smoothly, and the patient regained consciousness with no apparent neurological deficits.

Immediate post-operative head CT scan revealed no intracerebral bleeding or other abnormal conditions (Figure 1J). The patient experienced complete resolution of her preoperative trigeminal neuralgia. Postoperatively, she reported only mild, well-tolerated incision pain requiring no analgesic medication, with stable cognitive status and no decline in MMSE score. Transient pyrexia occurred but resolved promptly with standard supportive measures. She was discharged on postoperative day 8 in satisfactory condition. Complete pain relief was evident by a NRS score of 0 at 6 h postoperatively and persisted thereafter. During five years of follow-up, symptoms never recurred; she remained symptom-free at 105 years of age.

Notably, at the 3-month postoperative follow-up, the patient visited the clinic in high spirits, explicitly stating that the minimally invasive surgery had thoroughly resolved her suffering. She emphasized this procedure had granted her the chance to enjoy a high-quality elderly life, fully justifying her decision to undergo surgery after carefully weighing risks and benefits.

## Discussion

As the disease progresses, TN often transitions into a refractory stage, where even multimodal pharmacological regimens frequently fail to control symptoms, leaving the patients with no hope other than surgical management (9). MVD is the standard effective surgical treatment for TN. New research suggests that elderly patients undergoing MVD for TN can not only achieve a similar immediate pain relief rate but also demonstrate better long-term efficacy compared to their younger counterparts (10). Notably, there were no significant differences in the incidence of complications such as intracranial hemorrhage, bacterial meningitis, cerebrospinal fluid leakage, facial hypoesthesia, hearing impairment, and facial paralysis between the two age groups (3, 4, 10). Nonetheless, it's important to mention that the average age of elderly patients included in these studies typically revolves around 70 years (7, 10), with limited representation from the 80–90 age (4), even rarer cases exceeding 90, and no reported instances involving centenarians.

E-MVD leverages advanced endoscopic technology to offer a clearer and wider surgical field of view, empowering surgeons to accurately identify offending vessels and perform decompression procedures with greater precision and effectiveness. In contrast to traditional MVD, which often requires retracting the cerebellar hemisphere for a direct and tubular view, E-MVD eliminates this step, minimizing potential brain injuries associated with retraction. Thanks to its precise diagnostic capabilities and minimally invasive nature, E-MVD maximizes treatment efficacy while minimizing complications, ultimately expediting patients' post-operative recovery (5–8). Given these advantages, we opted for E-MVD to treat TN in this carefully selected centenarian patient. This approach achieved remarkable success, resulting in complete pain relief with no recurrence over five years. This landmark case underscores the potential of E-MVD as a viable and promising treatment option for TN in the extreme elderly, offering valuable insights for neurosurgeons globally.

However, it is crucial to acknowledge the inherent elevated risks associated with major surgery in this patient population. Centenarians typically exhibit reduced physiological reserve, increased frailty, and heightened susceptibility to complications such as cardiopulmonary events, delirium, and slower wound healing. The high prevalence of comorbidities and physiological decline in this patient population necessitates a rigorous, multidisciplinary preoperative evaluation. This assessment must focus on biological age, functional status, cognitive reserve, comorbidities, and patient goals of care, alongside meticulous anesthetic planning for hemodynamic stability. A tailored and individualized approach is essential to ensure optimal outcomes.

While this case demonstrates the feasibility and excellent long-term outcome of E-MVD in an ultra-elderly patient, it underscores that chronological age alone should not preclude intervention—patient selection is paramount. This study highlights E-MVD's potential as a viable treatment option even in centenarians with TN. Nevertheless, further research is needed to confirm these findings and explore the generalizability of this approach.

## Data Availability

The original contributions presented in the study are included in the article/Supplementary Material, further inquiries can be directed to the corresponding authors.
